# Fabrication and Characterization of a SPR Based Fiber Optic Sensor for the Detection of Chlorine Gas Using Silver and Zinc Oxide

**DOI:** 10.3390/ma8052204

**Published:** 2015-04-28

**Authors:** Sruthi P. Usha, Satyendra K. Mishra, Banshi D. Gupta

**Affiliations:** Department of Physics, Indian Institute of Technology Delhi, New Delhi 110016, India; E-Mails: sruthiprasoodu59@gmail.com (S.P.U.); satyendramishraiitd@gmail.com (S.K.M.)

**Keywords:** chlorine gas, optical fiber, sensor, surface plasmon resonance, zinc oxide

## Abstract

A fiber optic chlorine gas sensor working on surface plasmon resonance (SPR) technique fabricated using coatings of silver and zinc oxide films over unclad core of the optical fiber is reported. The sensor probe is characterized using wavelength interrogation and recording SPR spectra for different concentrations of chlorine gas around the probe. A red shift is observed in the resonance wavelength on increasing the concentration of the chlorine gas. The thickness of the zinc oxide film is optimized to achieve the maximum sensitivity of the sensor. In addition to wavelength interrogation, the sensor can also work on intensity modulation. The selectivity of the sensor towards chlorine gas is verified by carrying out measurements for different gases. The sensor has various advantages such as better sensitivity, good selectivity, reusability, fast response, low cost, capability of online monitoring and remote sensing.

## 1. Introduction

Chlorine gas, known as bertholite, is a highly virulent halogen gas with a reeking smell. It is being used for many applications such as bleaching agent, disinfecting agent, oxidizing agent, in producing pharmaceutical drugs, as combating putrefaction and even as a minacious weapon in wars. Apart from these applications it is harmful and adversely affects the environment and human beings. It causes irritation and oxidative injury to lungs, eyes, skin and even causes strangulation at higher concentrations. It is a corrosive gas that interacts with the human body in such a way that if it gets inhaled it would react with the moisture in the mucous membrane resulting in the formation of toxic hydrochloric acid and hypochlorous acid [1]. The chlorine gas would not sour as it is denser than air, hence would be difficult to dodge. In literature many gas sensors have been reported especially for the chlorine gas, which is of interest in the current paper, using electrochemical methods with ionic and semiconductor sensing elements [2,3,4,5,6,7,8,9]. These sensors have one or more of the following shortcomings: the methods are bit complicated, time taking, temperature dependent, high recovery time, needs to reduce oxygen deficiencies in case of metal oxide substrate such as zinc oxide, less reusability and more costly. One of the chlorine sensors reported has used zinc oxide with some surface modification to reduce the oxygen deficiencies as it makes the sensor insensitive to chlorine gas in its pure form [7]. The idea behind this sensor was making it sensitive by using surface additives to metal oxides. In the proposed chlorine sensor, zinc oxide has been used without any surface modification by applying the SPR technique making it highly sensitive to chlorine gas at room temperature.

Metal oxides have got a diversity of applications in the industrial fields such as environmental industries, oil and petroleum industries, chemical and physical industries and electronic and electrical industries. Since the metal oxides are prone to atmospheric factors such as temperature, pressure and the interacting gases, the molecular structure of the surface of metal oxides are different from that of the bulk. Mainly the surface and the sub-surface regions of metal oxides are included in reactions with either gases or liquids. Choosing a particular material for sensing of a particular gas depends on the interaction of its surface active sites formed by the ions O^−^, O^2−^, H^+^ and OH^−^ ions with the gas molecules. An advantage of metal oxides is that these can interact with both reducing as well oxidizing gases for its sensing. Zinc oxide is a post transition metal oxide and is surface conductometric. The characteristic property of zinc oxide which makes it useful for gas sensing is its reversible interaction with the gases due to covalent bond formation or dipole-dipole interaction [10]. The ejection of the gas out of the chamber along with the nitrogen purging leaves the zinc oxide sensing layer intact, as in the primal form, ready for further sensing. Further, zinc oxide is a semiconductor of n-type mainly due to intrinsic defects. When the sensing gas interacts with the metal oxide, adsorption of gas molecules at the surface of metal oxide takes place which changes its optical properties. The adsorption is decided by the presence of oxygen and hydroxyl ions on the zinc oxide surface. Such kind of a semiconductor metal oxide based gas sensor has got applications in many areas such as monitoring the environment quality, safety applications, quality affirmation, instrumentation and computations [11,12,13].

Surface plasmon resonance (SPR) is a promising technique that has unveiled many pronouncing applications in the field of optics, defense, communications, industrial and commercial products. Of these, the class of sensors forms one of the sizeable enactments. A metal-dielectric interface that supports the charge density oscillations is an inevitable condition. Surface plasmons can be defined as these charge density oscillations propagating through the metal-dielectric interface and the quantum of such oscillations are called surface plasmon [14,15,16,17,18,19]. To excite surface plasmons, a p-polarized light, since these are TM polarized in nature with an exponentially decaying electric field in metal as well as in dielectric medium, is required. This is accomplished under the resonance condition in which the energy is transferred to the surface plasmons from the evanescent wave (of the excitation light incident on the interface of a high refractive index medium and the metal film) which has the same wave vector as that of the surface plasmons. The particular angle of incidence at which the equality of wave vectors is satisfied is known as resonance angle. In the case of prism based SPR sensors, the measurement of the resonance angle for a given dielectric medium in contact of metal film is carried out using angular interrogation method. But in the case of fiber based SPR sensors, spectral interrogation method is used where the measurement of the resonance wavelength is performed [20,21,22,23,24,25,26,27]. The SPR spectrum of the polychromatic light launched in the fiber with SPR probe received at the output end of the fiber will have a dip corresponding to the dielectric constant of the medium surrounding the metal layer coated over unclad core region of the fiber. The wavelength corresponding to dip is called as the resonance wavelength and changes as the dielectric constant of the dielectric medium in contact of metal layer changes.

In this study we present the fabrication and characterization of a fiber optic sensor for the detection of chlorine gas using surface plasmon resonance technique. The probe is fabricated by coating an unclad core of the fiber by a thin film of silver followed by a film of zinc oxide. A feasibility/preliminary study of such kind of probe for the detection of chlorine gas was reported by us recently [28]. The present study deals with the more detailed rigorous study of the probe. For the characterization of the probe, light from a polychromatic source is launched from one end of the probe and the spectrum of the transmitted power at the output end of the fiber is recorded for a given concentration of the chlorine gas around the probe. The gas molecules interact with the zinc oxide layer and change its dielectric constant. The change in dielectric constant changes the resonance wavelength in the SPR spectrum recorded. To achieve the maximum shift in resonance wavelength for a given range of chlorine gas concentration the thickness of zinc oxide layer is optimized. Further, the selectivity of the probe for chlorine gas is checked by performing experiments with other gases.

## 2. Experimental

### 2.1. Fabrication of Probe

**Figure 1:**
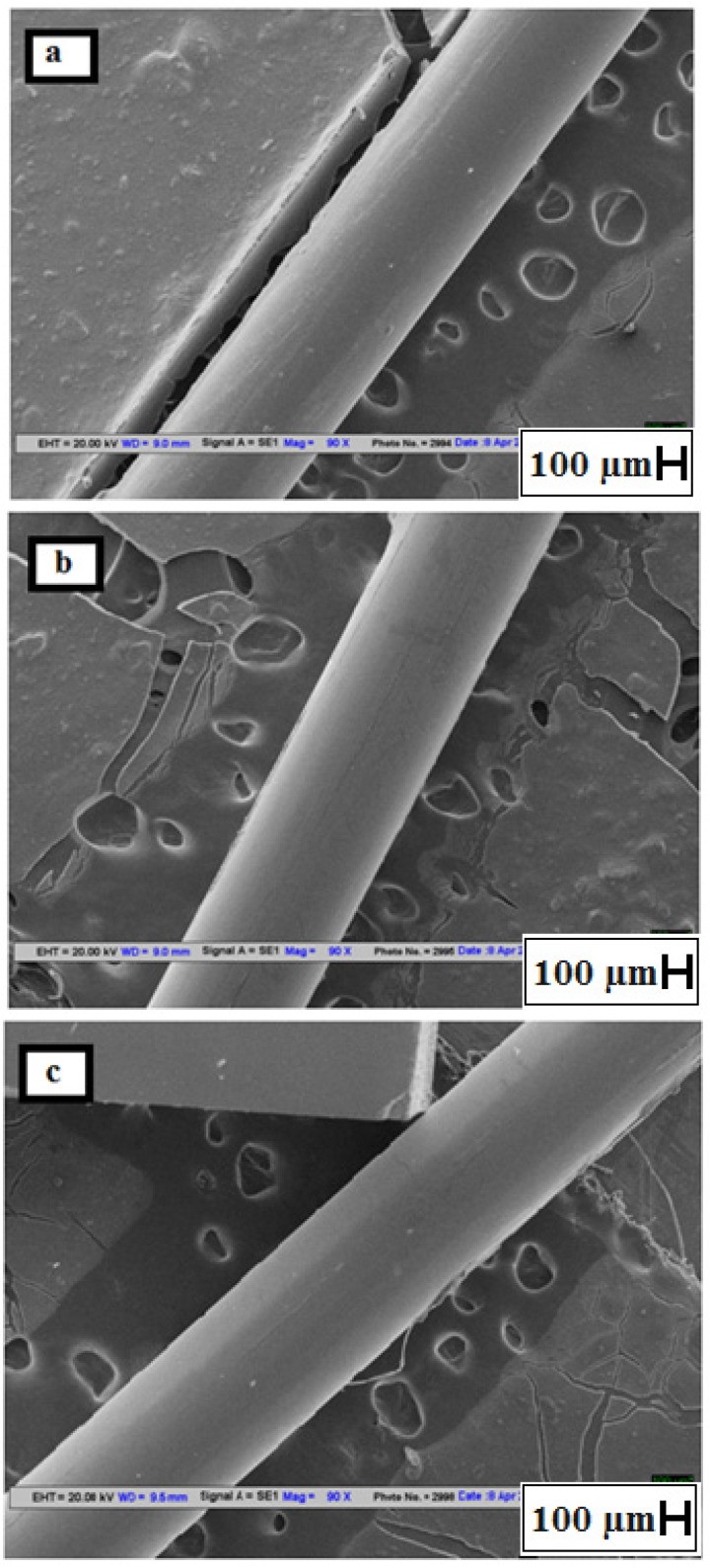
SEM images of (**a**) the unclad portion of bare fiber; (**b**) the silver layer coating on unclad fiber; (**c**) the zinc oxide layer coating on top of the silver layer coated unclad fiber.

For the fabrication of the probe plastic clad silica fiber having 0.40 numerical aperture and core diameter of 600 µm was used. Around 1 cm length of the cladding was removed from the middle portion of about 22 cm long fiber. The unclad core was cleaned with Millipore water and acetone before coating. It was first coated with 40 nm thick film of silver using thermal evaporation vacuum coating unit with a vacuum pressure of 5 × 10^−6^ mbars followed with coating of zinc oxide layer. A range of silver coating of 37–48 nm has been reported as the optimized thickness in the literature [29] and we opted 40 nm thickness of silver, while the optimized thickness for zinc oxide is not available and hence a number of probes were prepared with 40 nm thick film of silver but varying thicknesses of zinc oxide film over it. The thicknesses chosen for zinc oxide thin film were 15 nm, 18 nm, 20 nm, 25 nm and 30 nm. The coatings of plasmonic metal and metal oxides layer were performed using a thermal evaporation coating unit that operates in high vacuum. A quartz crystal has been arranged in the chamber of the coating unit to monitor the thickness when the coating is on. The thickness monitor has an accuracy of 0.1 nm. The quartz crystal thickness monitor attached with the coating unit was calibrated using the method of ellipsometry. The quality of the coatings was checked by using SEM (scanning electron microscope) images of the films. Figure 1a–c show the SEM images of the bare core (unclad) fiber, silver coated unclad fiber, and the zinc oxide coating over silver coated fiber respectively. The uniformity of the coated films can be seen from the SEM images. The surfaces have smooth texture without pores. Zinc oxide layer coated over silver layer in Figure 1c shows the smooth surface which confirms the uniform distribution/deposition of zinc oxide layer.

### 2.2. Experimental Setup

**Figure 2:**
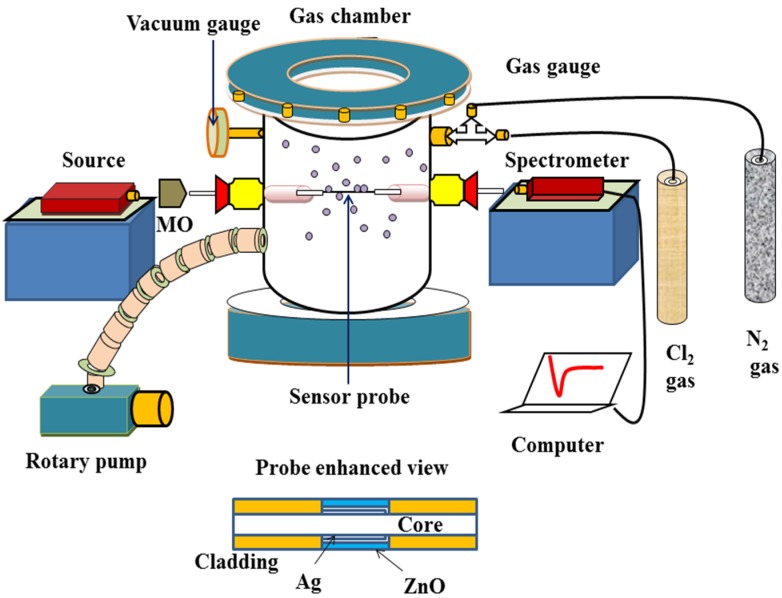
Schematic of experimental chlorine gas sensing set up.

The schematic diagram of the experimental set up is shown in Figure 2. The main part is the metal chamber which has the provision for inlet and outlet of the gas in addition to the ports for insertion of fiber optic probe. The chamber is kept airtight by using rubber septum. Before starting measurements, the vacuum was created inside the chamber by using rotary pump attached to it. Two cylinders were connected to the gas chamber, the one filled with chlorine gas to be sensed and the other filled with nitrogen gas used for purging. The pressure of chlorine gas and hence the concentration inside the chamber was measured using an already calibrated vacuum gauge attached with the chamber. The passage of the gas was adjusted by using appropriate knobs connected to the cylinders to the chamber. All the experiments were performed at room temperature and were carried out for different concentrations of chlorine gas ranging from 10 ppm to 100 ppm. Light from a tungsten halogen lamp (Model: AvaLight-HAL; Avantes, the Netherlands) was launched into the fiber and a spectrometer (Model: AvaSpec-3648; Avantes, the Netherlands) was used at the output end of the fiber to record the SPR spectrum by interfacing it with the computer for a given concentration of the gas in the chamber. The light source and the spectrometer were purchased from Avantes. The light from the source was effectively coupled into the fiber using a microscope objective and the light exiting from the other end of the fiber was directly fed into spectrometer to record the spectrum.

## 3. Results and Discussion

### 3.1. Sensing Principle

The sensing principle is based on the reversible chemical reaction which can be explained as follows. When chlorine gas is passed in the chamber it reacts with the zinc oxide thin film layer over the probe resulting in the formation of zinc chloride and oxygen [7,28].
2Cl_2_ (g) + 2ZnO(s) 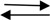 2ZnCl_2_ (s) + O_2_ (g)
(1)

In the case of SPR based sensors, the fundamental of sensing is based on the change in depth and shift in the SPR curves recorded corresponding to the change in the parameter of the analyte. In the case of gas sensing, as the concentration of the analyte gas changes, the shift and the depth of the dip of the spectrum change. These changes are in fact due to the changes in the real and imaginary parts of the dielectric constant of the sensing layer [10,15,17,18,19,21,22,23,24,25,26,27,28]. In the present study, the formation of zinc chloride results in the change in the dielectric constant/refractive index of the layer. As the change in refractive index increases, the shift in SPR curve increases. Along with the shift in SPR curve there can be an increase in the depth of the SPR curve with the increase in the concentration of the gas. This will occur due to the change in the imaginary part of the dielectric constant of the film along with the change in the real part which is responsible for the shift in the resonance wavelength. Thus, when both the resonance wavelength and the depth of the SPR curve are increasing this implies that both the real and imaginary parts of the dielectric constant of the zinc oxide layer are increasing on its reaction with the chlorine gas [15]. In the case of surface conductometric metal oxides such as zinc oxide, the adsorbed oxygen species plays an important role in reactions with the oxidizing and reducing gases. In the proposed sensor, the analyte interacts with the chemisorbed oxygen resulting in the formation of dipoles at the surface of the semiconductor metal oxide. This affects the free charge carrier concentration which results in the band bending. The adsorbed oxygen species on the surface conductive metal oxide such as ZnO causes an upward band bending and the analyte gas reacts with these pre-adsorbed oxygen species and results in the charge transfer [11,12,30]. This change in concentration of the charge carriers causes a change in the field or it may affect the “k” vector of the surface plasmons which in turn result in the shift in the resonance wavelength of the spectrum [18].

### 3.2. Thickness Optimization of Zinc Oxide Layer

**Figure 3:**
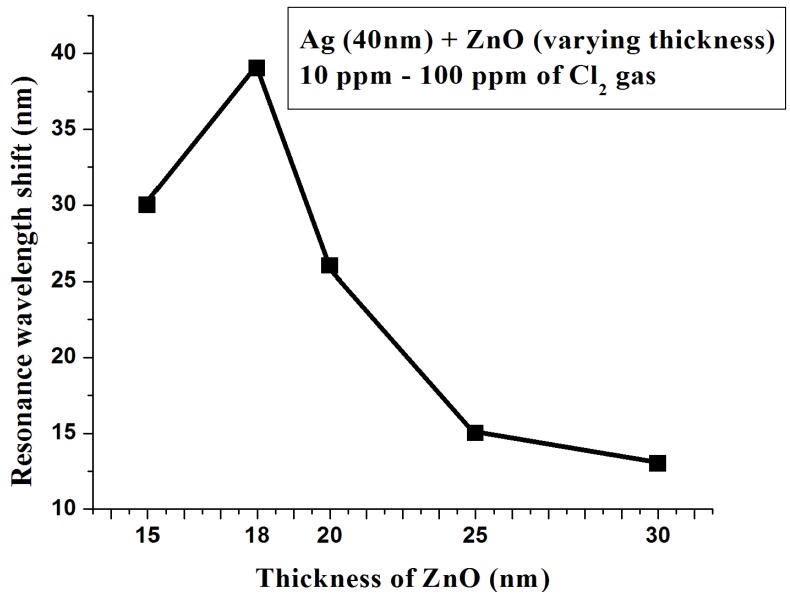
Shift in resonance wavelength as a function of thickness of zinc oxide thin film for the change in chlorine gas concentration from 10 ppm to 100 ppm.

The optimization of thickness of zinc oxide was an important fragment of this work that gives the maximum shift in resonance wavelength and therefore the maximum sensitivity. The thickness of the zinc oxide layer was varied as 15 nm, 18 nm, 20 nm, 25 nm and 30 nm. The probes were prepared with different thicknesses of zinc oxide layer on top of silver layer on the unclad core of the optical fiber. The experiments were conducted by using the set up shown in Figure 2. The SPR spectra were recorded for 10 ppm and 100 ppm concentrations of chlorine gas in the chamber and from these spectra shift in resonance wavelength were determined for probes with different thicknesses of zinc oxide layer. The shift in resonance wavelength for the change in chlorine gas concentration from 10 ppm to 100 ppm for different thicknesses of zinc oxide thin film is shown in Figure 3. It has been observed that as the thickness increases the shift in resonance wavelength improves initially. For thickness of 15 nm, a shift in resonance wavelength of 30 nm was observed and the resonance wavelength shift reached to a maximum value of 39 nm for 18 nm thickness of zinc oxide layer and as the thickness of the layer was further increased the shift in resonance wavelength got decreased and has encountered with the minimum shift in resonance wavelength for 30 nm thickness of the zinc oxide layer.

Thus the thickness of zinc oxide thin film for chlorine gas sensing is optimized as 18 nm since it gives the maximum shift in resonance wavelength of 39 nm. The maximum shift in resonance wavelength or the highest sensitivity for a particular thickness of the over layer occurs due to the thickness dependence of the electromagnetic fields distribution in a multilayer structure [31]. In the present probe, the evanescent field produced at the zinc oxide and chlorine gas medium interface is maximum for 18 nm thick film of zinc oxide.

### 3.3. Characterization of the Sensor Probe

**Figure 4:**
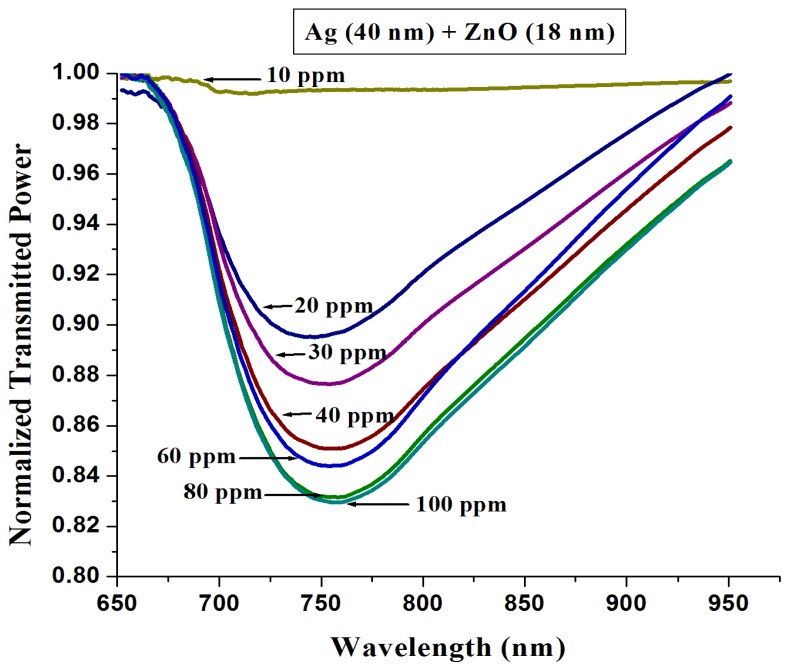
Normalized transmitted power spectra as a function of wavelength for different concentrations of chlorine gas.

To characterize the fiber optic probe for the chlorine gas sensing experiments were carried out for different concentrations of the gas inside the chamber and the SPR spectra were recorded. Figure 4 shows the obtained SPR spectra for varying concentration of chlorine gas from 10 ppm to 100 ppm for sensor probe with 40 nm thick silver coating and 18 nm zinc oxide thin film over unclad core of the fiber. It is clear from the plot that the SPR curve shifts towards the right side with red shift in the resonance wavelength. The reversible chemical reaction responsible for the shift in resonance wavelength has been already explained above.

**Figure 5:**
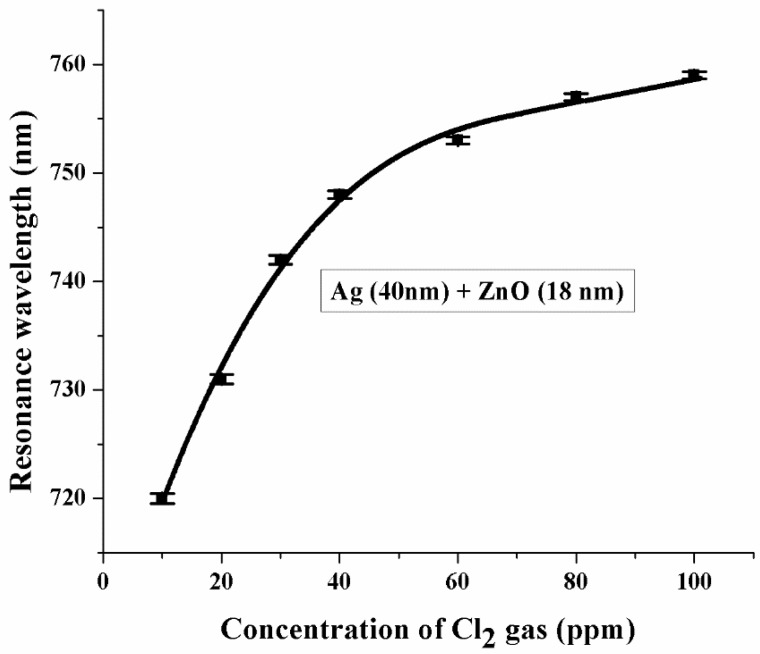
Variation of resonance wavelength as a function of concentration of chlorine gas.

The variation of resonance wavelength corresponding to the change in the concentration of the chlorine gas from 10 ppm to 100 ppm is shown in Figure 5. The error bar shown with each data point is the maximum possible error in the resonance wavelength that have occurred while performing experiments for the same concentration three times within a period of 30 days. For each evaluation the errors measured came within the error bars shown in the figure. This shows the repeatability of the probe and the robustness of the data acquired. It has been elucidated from the figure that the resonance wavelength has varied more at lower concentration and it also defines the operation range as the curve shows saturation behavior at higher concentrations. This process can be explained by the interaction of post transition surface conductive metal oxide, ZnO, having d^10^ configuration with gases [30]. Here the chlorine is acting as an oxidizing agent.

In the case of surface conductometric semiconducting metal oxides such as zinc oxide, the gas sensing mechanism is mainly based on the adsorption of the chemicals/ gas molecules directly on the metal oxide surface or by the reaction of the gas species with the surface adsorbed oxygen. As soon as a metal oxide coating is completed and when it is exposed to the atmosphere, the chemisorptions of oxygen molecules occur which participate in the redox reaction with the gas species. The reaction depends on the adsorption of the oxygen molecules on the metal oxide surface and the redox reaction that is happening between the chlorine gas and the active sites on the metal oxide [11,12,32]. The sensing data were acquired by passing pure chlorine gas of appropriate concentration into the enclosed gas chamber. The setup does not have a flow rate analyzer, but the chamber has the capability to keep the filled concentration intact which decides the corresponding spectrum shift and dip.

The trend of curve in Figure 5 can be explained using the concept of active site. At lower concentration since the active sites available on ZnO per gas molecule is more, the reaction will be more resulting in a large change in the dielectric constant and hence the large shift in the resonance wavelength. But as the concentration of chlorine gas increases, since most of the active sites have been occupied by the gas molecules, the number of active sites available per chlorine molecule for the reaction would be less resulting in less reaction which decreases the rate of increase of the shift of resonance wavelength. This explains the saturation behavior of the curve at higher concentrations.

**Figure 6:**
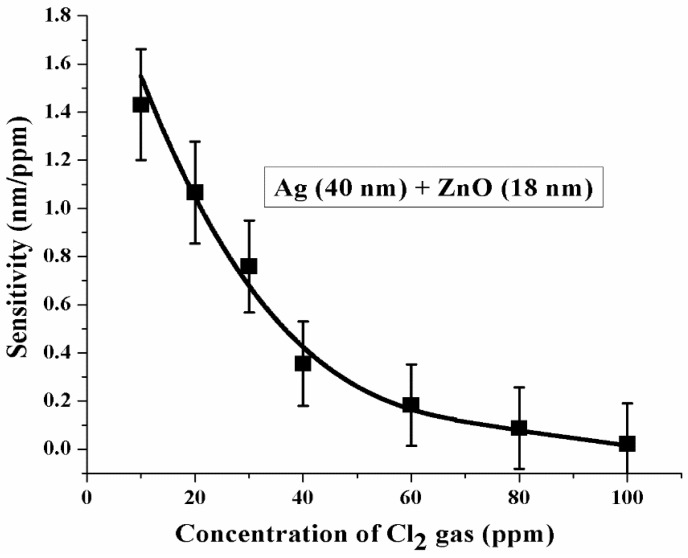
Variation of the sensitivity of the sensor probe with the concentration of the chlorine gas.

The plot of sensitivity with chlorine gas concentration in the range from 10 ppm to 100 ppm is shown in Figure 6. The sensitivity is defined as the change in resonance wavelength per unit change in the concentration of the gas and was determined, in the present study, by using a derivative procedure of the curve used to fit the response curve (Figure 5). The partial derivative of the resonance wavelength with respect to the concentration of chlorine gas equation was performed for each concentration by substituting the corresponding chlorine gas concentration in that equation [22]. The sensor shows a maximum sensitivity of 1.4 nm/ppm at 10 ppm concentration which steadfast the concept of maximum subtlety at low concentration. As the concentration increases, the reaction taking place between the chlorine gas molecule and zinc oxide thin film decreases which in turn decreases the sensitivity of the system hitting almost zero at 100 ppm.

**Figure 7:**
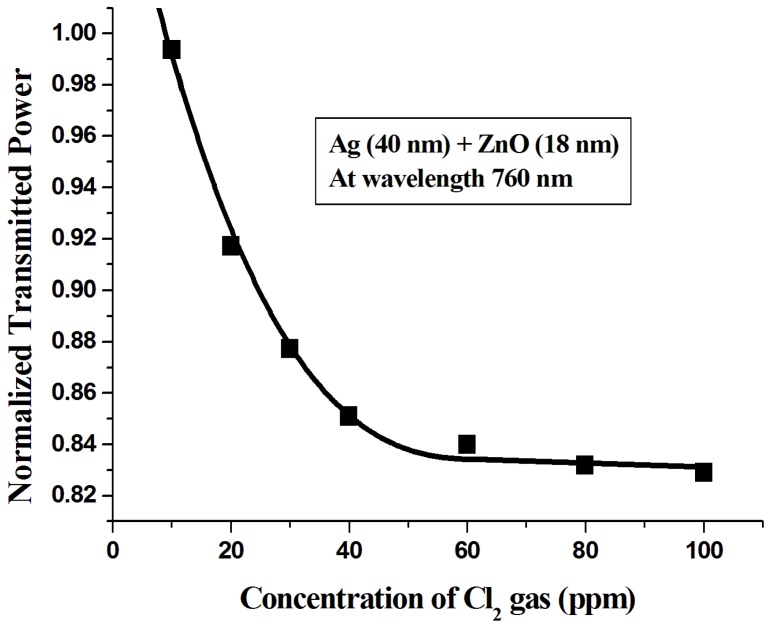
Variation of normalized transmitted power at 760 nm as a function of concentration of chlorine gas.

The normalized transmitted power for varying concentration of chlorine gas at a particular wavelength of 760 nm is shown in Figure 7 which shows the sensor calibration with intensity modulation. The reason of choosing 760 nm wavelength was that the resonance occurs around this wavelength for the chlorine gas concentration of 100 ppm. The normalized transmitted power variation with chlorine gas concentration is in agreement with the results plotted in Figure 5 on the variation of resonance wavelength. The sensitivity reaches to zero if intensity modulation, in place of wavelength interrogation, is used for the present sensor. The error bars shown in the figure are the maximum possible errors in the sensitivity for different concentrations of the chlorine gas.

**Figure 8:**
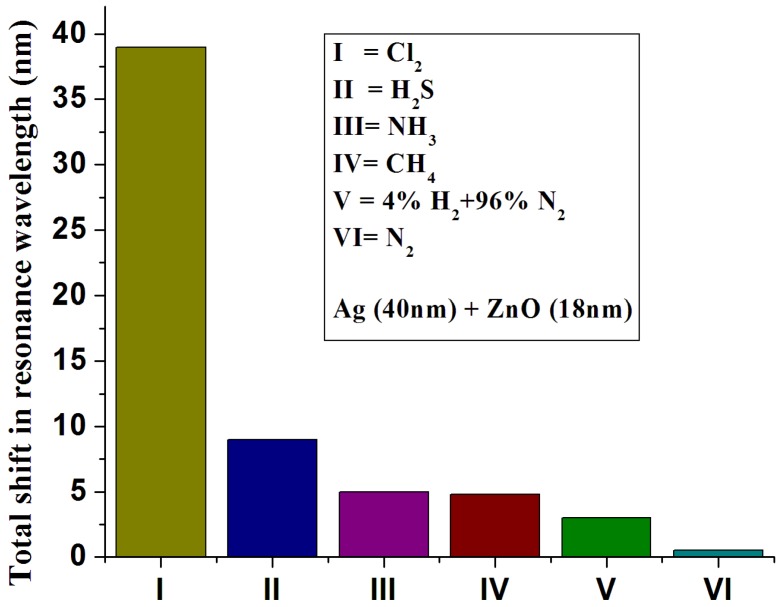
Total shift in resonance wavelength for the concentration change from 10 ppm to 100 ppm for different gases around the probe.

The study of selectivity of the analyte has also been carried out. The performance of the sensor was checked for different gases such as hydrogen sulphide, ammonia, methane, hydrogen and nitrogen and compared with that of the chlorine gas. Depending on the reaction of these gases with zinc oxide, chlorine acts as an oxidizing agent, whereas hydrogen sulphide, hydrogen, ammonia and methane act as reducing agents and nitrogen is an inert gas which is quite unreactive with the probe. The shift in resonance wavelength for the change in concentration of each gas from 10 ppm to 100 ppm was determined. The results so obtained are shown in Figure 8. It shows that the shift in resonance wavelength is very large for chlorine gas when compared to others, hence the fiber optic probe prepared with coatings of silver (40 nm) and zinc oxide (18 nm) gives the maximum sensitivity to chlorine gas and is the best combination. Further, it may be noted that all the gases showed a red shift in resonance wavelength in the recorded spectra with nitrogen as the least reacted or non-reacted analyte. For all the selectivity tests and main experiments conducted, the composition inside the metal chamber was the corresponding pure analyte only.

As mentioned above, to check the reusability of the sensor, experiments were carried out number of times on the same probe on different days. The results obtained were within a delta value of ±0.46 nm. Further the sensor has fast response. This was tested by recording the SPR spectrum at different times after the insertion of the gas of a given concentration in the chamber. No change in SPR spectrum was observed after 1 minute of insertion of the gas in the chamber.

## 4. Conclusions

In summary, chlorine gas sensing has been carried out using layers of silver and zinc oxide over unclad core of the fiber. The gas sensing depends on the interaction of metal oxide with the gas. To achieve the best performance of the sensor, the thickness of the zinc oxide film has been optimized. The optimized values of thicknesses of silver and zinc oxide layers give a shift of 39 nm in resonance wavelength for a concentration change of chlorine gas from 10 ppm to 100 ppm. The sensor can work both in spectral and intensity interrogations. Further, the selectivity test has been performed for upholding the selection of analyte. The sensor has various advantages such as high sensitivity, good selectivity, low cost and capability of online monitoring and remote sensing because the probe is fabricated on optical fiber.
